# The Golden Age in Cancer Nanobiotechnology: Quo Vadis?

**DOI:** 10.3389/fbioe.2015.00142

**Published:** 2015-09-22

**Authors:** João Conde

**Affiliations:** ^1^Harvard-MIT Division for Health Sciences and Technology, Institute for Medical Engineering and Science, Massachusetts Institute of Technology, Cambridge, MA, USA

**Keywords:** nanobiotechnology, cancer nanotechnology, nanomedicine, translational medical research, clinical trials as topic, *in vivo* models, medical devices

Since *Richard Feynman* and his famous talk “There’s plenty of room at the bottom” in an American Physical Society meeting at Caltech in 1959, Nanotechnology has led to the development of novel materials and devices with a wide-range of applications, especially in imaging, diagnostics, and therapy, which contributed to the early detection and treatment of cancer and metastasis (Ferrari, 2005; Conde et al., 2012a; Schroeder et al., 2012).

Although Nanotechnology is thought to be a new branch of Science that has only emerged over the past decade, nanoparticles (for example, gold nanoparticles) have been used, even though inadvertently, for several thousand years (Salata, 2004). In fact, these nanoparticles have been regarded as precious for as long as humans have existed, and have been associated from the time of gods and kings to the time of *Faraday* (Wagner et al., 2000; Edwards and Thomas, 2007).

Today, nanotechnology is a flourishing field that is helping to address critical global problems from cancer treatment to climate change. In fact, nanotechnology is everywhere and is everyday practice.

Nowadays, nanomaterials and nanoparticles have gained increasing interest due to their extraordinary electrical, optical, and chemical properties, high stability and biological compatibility, controllable morphology and size dispersion, and easy surface functionalization (Anker et al., 2008; Parveen et al., 2012; Conde et al., 2014a).

A unique feature of nanomaterials in the nanometer range (such as high surface-to-volume ratio or size-dependent optical properties) is that they are radically different from those of their bulk materials and with a huge potential to be used in the clinical field for disease diagnostics and therapeutics (Kim, 2007; Heath and Davis, 2008). The most common bioapplications in which nanomaterials and nanoparticles have been used so far are labeling, delivering, heating, sensing, and detection (Sperling et al., 2008), using several approaches, such as gene delivery, tumor targeting, or drug delivery, especially in cancer (Figure 1).

**Figure 1:**
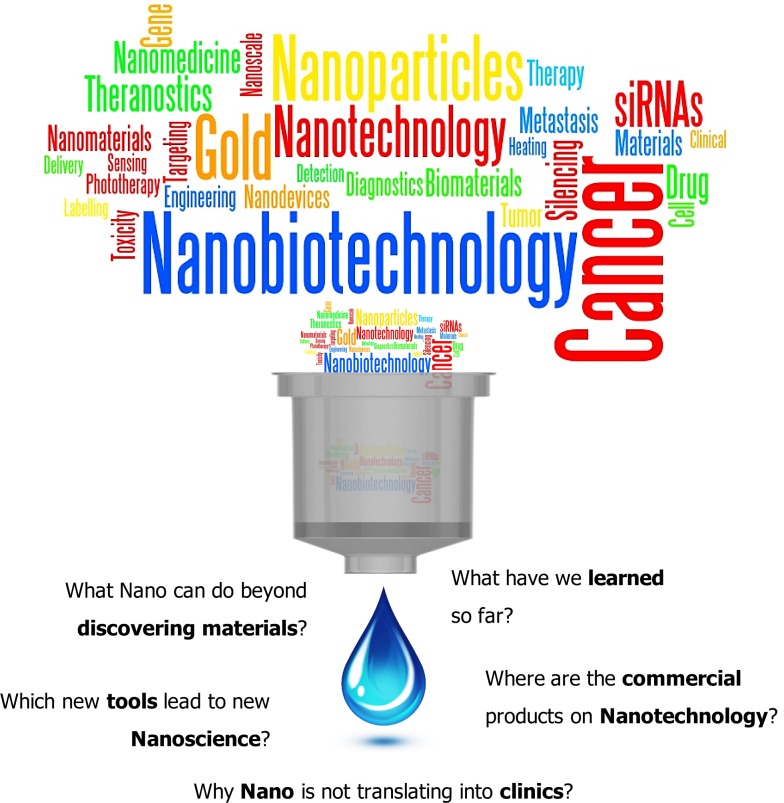
**The golden age in cancer nanobiotechnology: promises and pitfalls**. After a decade of substantial investment around the world, we are often asked what nano can do beyond discovering new systems and methods to produce novel materials? Although all studies described so far provide a baseline level of data in support of the effectiveness and safety of nanomaterials, we wonder what have we learned so far? Which new tools lead to new Nanoscience? Where are the commercial products on Nanotechnology? Is nanotechnology truly the next trillion-dollar market?

Cancer is the one of first leading causes of mortality worldwide, with more than 14 million new cases and 8.2 million cancer-related deaths only in 2012. According to the National Cancer Institute, in 2015, an estimated 1.5 million new cancer cases will be diagnosed and almost 600,000 cancer deaths will occur only in the United States (Siegel et al., 2014). The three most common cancers in 2015 are projected to be breast cancer, lung and bronchus cancer, and prostate cancer. And the problem is far away from getting better. The global cancer rates could increase by 50% to 15 million by 2020, according to a report from the World Health Organization. And the national costs for cancer care in the United States totaled nearly $125 billion in 2010 and could reach $156 billion in 2020.

Although we have assisted in the last 50 years to rapid technological advances, the full understanding of the molecular onset of this disease is still far from being achieved, as well as the search for new mechanisms of treatment that rely on selectivity and specificity toward cancer cells only (Peer et al., 2007). It is here that nanotechnology enters the fight offering a prosperity of utensils for cancer diagnostics and therapy. The main challenge is to develop a system capable of circulating in the blood stream undetected by the immune system or being applied locally for sustained release of therapeutic payloads, able to identify the required target and signal it for efficient and specific delivery (Conde et al., 2014b). As a result, nanotechnology is playing a pivotal role in providing new types of nanotherapeutics for cancer that have the potential to provide highly active and specific therapies with minimal side effects and maximum therapeutic outcome. Smart and multifunctional nanomaterials are one of those nanosystems that represent non-toxic and multipurpose mediators for a variety of biomedical applications, such as diagnostic and imaging assays (Cao et al., 2005; Conde et al., 2010; Kim et al., 2011), phototherapy and radiotherapy improvement (Hainfeld et al., 2008; Oh et al., 2014), as well as tumor targeting (Conde et al., 2012b, 2013, 2014c), gene silencing (Conde et al., 2012b, 2015a,b; Jiang et al., 2013), and drug delivery (Gibson et al., 2007; Han et al., 2007; Lai et al., 2007; Conde et al., 2015b).

This is feeding the biopharmaceutical companies to develop and expand the pool of potential therapeutic targets. The growth of this target pool will help overcome the already precarious bottlenecks in drug discovery pipelines, as the key steps for validating targets as legitimate candidates for therapeutic intervention. Nanotechnology will undoubtedly be crucial for identifying useful drug target candidates and for validating their importance and efficacy in disease states, such as cancer (Conde et al., 2015a). Actually, cancer nanotechnology may offer new opportunities for personalized oncology in which diagnostics and therapy are based on each individual’s molecular and cellular profile (Ferrari, 2005).

In the last 30 years, thousands and thousands of nanosystems were published in peer-review journals. These systems will most likely modernize our understanding of biological mechanisms and push forward the translation to clinical practice. However, after three decades of substantial investment around the world, we are often asked what Nano can do beyond discovering new systems and methods to produce innovative materials? Although all studies described so far provide an important baseline level of data in support of the effectiveness and safety of nanomaterials, we wonder what have we learned so far? Which new tools lead to new nanoscience? Where are the commercial products of nanotechnology? Is nanotechnology truly the next trillion-dollar market?

At the first place, it is imperative to learn how advances in nanosystem’s aptitudes are being used to find new diagnostic and therapy platforms driving the expansion of personalized medicine in oncology. And most important to learn what approaches are proving fruitful results in turning promising clinical data into treatment realities (Conde et al., 2014b).

A recurrent problem that we can find in literature is that the majority of studies on nanomaterials do not consider the final application to guide the design of nanomaterials. Instead, the focus is predominantly on engineering materials with specific physical or chemical properties (Albanese et al., 2012). Besides, we are still considering numerous studies reporting *in vitro* cell studies only. Despite the significant efforts toward the use of nanomaterials in biologically relevant research, more *in vivo* studies are needed to assess the applicability of these materials as delivery agents. Future *in vivo* work will need to carefully consider the correct choice of chemical modifications to incorporate into the multifunctional nanocarriers to avoid activation of off-target and side effects, as well as toxicity. Although is true that clinical trials using nanomedicines are not very common, most researchers do not seem to take benefit of the published studies in clinical trials to guide the design of new materials. Published trials with patient data can guide and focus researchers in their study goals, as well as to achieve and improve long-term positive consequences for the growth and expansion of the nanotechnology field. Therefore, it is extremely encouraging for academia and industry to publish nanotechnology-based clinical trials in nanotechnology and materials journals, even the ones with negative results. These data would serve as a foundation for measuring real clinical value and to develop stronger and reliable protocols in future clinical trials.

In the absence of a future proactive intervention, we take the risk that nanomedicine studies founded on limited clinical translation may discourage not only public confidence but also more importantly drive funding to other fields of research, which are supposed to engender more valuable results.

So why the majority of the manuscripts published in top nanotechnology journals with new nanomaterials do not report on clinical data to validate their findings? Although we know that maintaining animal set-ups is expensive and time consuming and that sponsoring clinical trials may cost millions of dollars, a greater problem may also rely on the genesis of most researchers training skills. Most of the nanotechnology research teams are trained in physics, chemistry, and/or engineering and lack the bridge between technology and clinics. And the opposite also occurs clinicians classically do not tend to endeavor into nanotechnology research. This may explain the disproportional level between nanomaterials production and their translation into clinics. Only a very small portion of all nanomaterials were produced to improve a bench-to-bedside approach to translational research. Outcomes like this must be followed by extensive laboratory work, which results in improved screening procedures and a new therapy of great potential, although the final product should always be part of a two-way interaction between laboratory scientists and clinicians. With chemists, biologists, and materials scientists working together with clinicians and engineers, but especially with “translational entrepreneurs” new solutions to crucial nanobiomedical problems will hopefully be found (Conde et al., 2014b).

Another important question is that after billions of dollars of investments in nanoscience and nanotechnology around the world we are often asked: where are the commercial products on nanotechnology? and is nanotechnology the next trillion-dollar market?

In fact, the National Science Foundation estimates that the global nanotechnology market could be valued in $1 trillion by 2015, and expected to be a fundamental driver of job creation in the years and decades ahead. This occurs once nanotechnology is now entering sectors as diverse as healthcare, auto manufacturing, and food production. A key driver of market development is the “acceptance” and “adoption” of nanotechnology by the main industries.

However, these numbers are more reminiscent of supermarket tabloids than serious market research, once many of the innovative and disruptive technologies have failed to emerge. Certainly, 7 years from the beginning of the National Nanotechnology Initiative, there appears to be a diminutive sign of a nanotechnology-based industry, although important amounts of R&D are being assumed by several industries. We are in danger of letting the commercialization of nanotechonology at the hand of speculative and science fiction tabloids (Weiss, 2015). In fact, developing commercial products may take several years and millions of dollars in order to progress and overcome the “Valley of Death,” which usually overlaps with the phase when the company receives an initial capital contribution to begin generating revenues. Since during this phase, the additional financing is scarce, the company may become vulnerable to cash flow requirements. The only way to overcome this period is to have a “champion product that carries the trophy,” bridging the “Valley of Death.” A successful outcome usually requires a strategy based on the knowledge of potential resources, leadership in attaining the resources and passionate dedication of staff to executing the development plan.

Above and beyond, numerous “barricades” stand between verification of efficacy and safety, ethical issues, as well as regulatory approval by the Food and Drug Administration (FDA), making it extremely difficult to advance to commercialization, and make it to and through clinical trials and patients at the end. In fact, the turnover of the “commercialization” or the proof of concept occurs only after spending huge amounts of money and time. If a specific product is able to go through the “Valley of Death” with success the probability for reaching the market is higher. But this process may take as long as 20 years (Conde and Artzi, 2015). Usually in the first year, we may have ~10,000 leading discoveries that will drop down into ~250 after safety and efficacy testing in animals in the following 2–4 years. Between year 6 and 10 this number decreases to ~10 after human clinical trials and to 1 at the end of 15 years following regulatory approval. To further hamper this process, after regulatory approval, ~70% of the products need additional studies.

For nanotechnology developments to range their full potential, the FDA must play a progressively vital role as an agency dedicated not only guaranteeing safe and active products but also to encourage and contribute more aggressively in the scientific and technological research initiative directed toward new nanotechnology-based platforms. In fact, more than 30% of the new medical products recently approved by the FDA are combination of products previously approved. Intense debates about the safety of nanoscale science are likely to endure for many years until a sufficiently strong foundation of applications in the field exists on which to base long-term decisions. For this reason, we have and must to modernize our evaluation and approval processes to guarantee that ground-breaking products reach the patients who need them, when they need them.

We know that test protocols must run according to established standards for drugs, as well as for device biomaterials, components, and finished products. However, since living tissues behave differently between nano and macro structures, unique test protocols must be developed. Possibly, expenditures experienced in qualifying new, potentially “disruptive” nanotechnology-based medical products may exceed those of conventional products.

In summary, the major recommendations to the field pass intensively trough the increase in support the commercialization of nano products, including nano manufacturing, ensuring not only the support of translation science as well as medicine and engineering but also a better coordination between the involved agencies (Weiss, 2010).

Increased efforts in nanoscience tutoring and in understanding the social impacts of nanotechnology are also of utmost importance. In fact, one of the first challenges may be to raise consciousness and instruct the community on nanotechnology and its potential impact.

Another key reference is the fact that novel nanoscale tools enable insight and awareness, smart answers, and original questions, leading to a biotechnology revolution, raising our efforts in nanoscience and nanotechnology.

Last but not least, it is essential that governments, Institutions, and Funding Agencies support selected fields of science and technology based on the acknowledgment of the possible reimbursements or advances for society, in order to accelerate the development of the field.

An active approach, which consciously support research at interfaces between materials and medicine (i.e., nanomedicine), will require systemic changes, as well as new funding mechanisms. National or international funding agencies should foster changes in the R&D support systems, by realizing the pioneering potential of such an interdisciplinary field as nanotechnology. Moreover, to monitor the previously recognized trends and gradually adjust them as a request that the R&D community imposes may be the key of a reliable, sustained, and golden age of nanobiotechnology.

## Conflict of Interest Statement

The author declares that the research was conducted in the absence of any commercial or financial relationships that could be construed as a potential conflict of interest.
